# Habitat preference of blackflies in Omo Gibe river basin (southwest Ethiopia): Implications for onchocerciasis elimination and control

**DOI:** 10.1371/journal.pone.0264750

**Published:** 2022-03-04

**Authors:** Beekam Kebede Olkeba, Seid Tiku Mereta, Peter L. M. Goethals, Delenasaw Yewhalaw, Gemechu Debesa, Argaw Ambelu, Mahmud Ahmednur, Pieter Boets

**Affiliations:** 1 Department of Animal Sciences and Aquatic Ecology, Ghent University, Ghent, Belgium; 2 Department of Environmental Health Science and Technology, Jimma University, Jimma, Ethiopia; 3 Department of Environmental Health Science, Hawassa University, Hawassa, Ethiopia; 4 School of Medical Laboratory Sciences, Jimma University, Jimma, Ethiopia; 5 Tropical and Infectious Disease Research Center, Jimma University, Jimma, Ethiopia; 6 Department of Geography and Environmental Studies, Jimma University, Jimma, Ethiopia; 7 Provincial Centre of Environmental Research, Ghent, Belgium; University of Eldoret, KENYA

## Abstract

Ecological control of blackflies (*Simulium damnosum*) can be an alternative or additional tool to enhance onchocerciasis elimination efforts. However, limited research is conducted on the ecology of blackflies in Ethiopia. In this study, we determined the habitat preference of blackfly larvae and their relationship with aquatic macroinvertebrate predators in the Omo Gibe river basin of southwest Ethiopia. Environmental and biological data were collected from 150 sampling sites during both dry and wet seasons in 2019. Generalized Linear Models (GLMs) were used to identify factors affecting the occurrence and abundance of *S*. *damnosum* larvae. Canonical Correspondence Analysis (CCA) was used to investigate the relationship between environmental and biological variables and the abundance of *S*. *damnosum* larvae. The findings of this study indicated the abundance of *S*. *damnosum* larvae increased with increasing turbidity, alkalinity and altitude, but decreased with increasing concentrations of five-day Biological Oxygen Demand (BOD_5_), orthophosphate and magnesium ion. Both the presence and abundance of *S*. *damnosum* larvae decreased with the increasing abundance of stonefly larvae (Perlidae). *Simulium damnosum* larvae were found less likely in the presence of mayfly larvae (Baetidae) and were less abundant where Chironomidae are abundant. In conclusion, the findings of this study showed that the habitat preference of *S*. *damnosum* larvae is determined by environmental factors and that the presence and abundance of the larvae are affected by macroinvertebrate predators. It is essential to establish buffer zones as a part of watershed management to retain pollutants and prevent them from entering directly into water courses to improve water quality and the assemblages of macroinvertebrate predators and enhance biocontrol of blackflies.

## Introduction

Blackflies are insects of medical and veterinary importance whose immature stages are exclusively aquatic [1]. The family Simuliidae comprises about 26 genera and more than 2000 species, most of which are hematophagous [2]. Simuliids have a holometabolous development cycle [3]. The immature stages of blackfly inhabit lotic water bodies which are the sessile filter-feeding larvae and they are often forming a large proportion of the benthic biomass [3, 4]. They are confined to well-oxygenated sections of streams, rivers, waterfalls and spillways since adult fly prefers these habitats for laying eggs and in addition, it is a suitable environment for larval development [5]. There is a worldwide distribution of simuliid and the taxon is found in most rivers extending from the tropics to the Arctic Circle, even in desert ecosystems and in high polar latitudes, and coral islands [6]. Simuliids are found attached to various substrates in freshwater streams and exhibit a peculiar selection of breeding habitats [7]. Adult female blackflies require a blood meal to reproduce [8]. In view of this, human settlements close to rivers and streams are usually more affected by the biting nuisance and infection [9, 10].

Adult female blackflies of the genus *Simulium* are known for transmitting a filarial worm *Onchocerca volvulus*, the causative agent of onchocerciasis or river blindness [11]. In Ethiopia, two blackfly species have been identified to be vectors of *O*. *volvulus*. *Simulium damnosum* sensu lato (s.l.), the major vector of *O*. *volvulus*, has a wide distribution throughout endemic and non-endemic areas of the country. *Simulium ethiopiense*, which has a restricted distribution in the smaller rivers of the southwestern midlands and the highlands and which is often sympatric with *S*. *damnosum* s.l., is suspected to play a secondary role [12]. Onchocerciasis is one of the most important neglected tropical diseases and it is the world’s second-leading infectious disease causing blindness, after trachoma [13]. Globally, an estimated 37.2 million people are infected with onchocerciasis and approximately 1 million people are blind or visually impaired and another 120 million people are at risk of infection [14]. It is estimated that 99% of the cases are found in Africa [15]. The disease is also endemic in Yemen and certain Americas countries [16]. In Ethiopia, about 20 million people live in the surveyed endemic areas and 3 million people are infected with onchocerciasis [17].

The Federal Ministry of Health of Ethiopia developed a Master Plan and Roadmap to eliminate onchocerciasis by the end of 2020 and to be certified free from onchocerciasis by 2025 [18]. Ivermectin is a safe and highly potent microfilaricidal drug that has assumed a major role controlling or eliminating onchocerciasis [19]. Despite the use of Ivermectin for many years, the transmission of onchocerciasis in many districts remained unabated [20]. The main challenge experienced in eliminating onchocerciasis is the possibility that Ivermectin resistance is emerging [21–23]. There are reports of *O*. *volvulus* responding poorly to the anti-fecundity effect of Ivermectin in Ghana [24, 25] and Cameroon [26, 27]. Therefore, there is a need for an alternative or additional tool to reduce the current reliance on the chemotherapeutic approach using Ivermectin for the successful elimination of onchocerciasis.

Ecological control of immature blackflies can be an alternative or additional tool to enhance onchocerciasis elimination efforts [28]. Therefore, knowledge of the ecology of target species is crucial to consider ecological control of immature blackflies as a component of an integrated onchocerciasis elimination program [5, 29]. Previous studies have reported that the habitat preference of blackflies is determined by a wide range of environmental factors including water temperature, pH, turbidity, dissolved oxygen, biological oxygen demand, electrical conductivity, total suspended solids, total dissolved solids, nitrate, orthophosphate, alkalinity, hardness, riparian vegetation cover, canopy cover, stream flow velocity, stream flow rate, altitude and others [5, 7, 29–37]. In addition, ecologists have revealed that biotic factors such as predation [38, 39] and competition [40] are important drivers of the blackfly population. Several aquatic macroinvertebrates in the Hemiptera, Trichoptera, Plecoptera, Odonata, Ephemeroptera and Coleoptera orders have been reported as potential biocontrol agents of blackfly larvae [29, 41–46]. The co-occurrence of blackfly larvae and their potential macroinvertebrate predators is what is expected of organisms that exercise mutual population regulation in aquatic environments [7, 47]. In this context, aquatic macroinvertebrate predators could be used as potential biocontrol agents of immature blackflies, implying this strategy can manage Ivermectin resistance to enhance onchocerciasis elimination efforts. Because of environmental heterogeneity among ecological regions, it is essential to understand the ecology of blackflies at the local level. Except for a few studies by Ambelu et al. [29], little is known about the ecology of blackflies in Ethiopia.

Therefore, this study aimed to determine the habitat preference of blackfly larvae and their relationship with aquatic macroinvertebrate predators in the Omo Gibe river basin of southwest Ethiopia. The findings of this study provide valuable information that can be used to identify preferred habitats by blackfly larvae and design biocontrol of blackflies as a potential alternative or additional tool to support the efforts to eliminate onchocerciasis.

## Methods

### Study area

**Fig 1:**
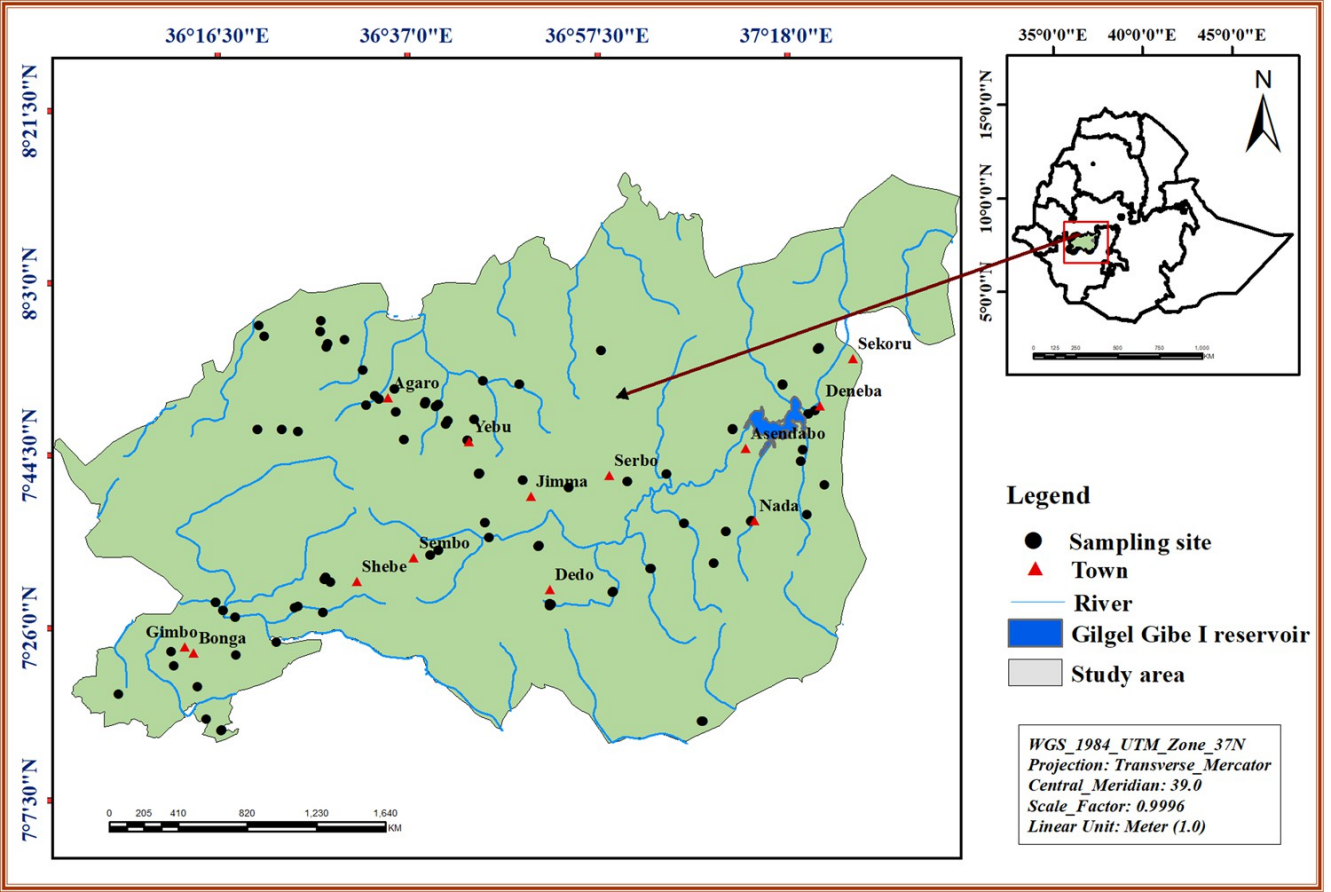
Map of the study area and locations of the sampling sites in the Omo Gibe river basin, southwest Ethiopia.

This study was conducted in the Omo Gibe river basin in southwest Ethiopia (**Fig 1**). The Omo Gibe river basin lies between latitudes 4°25′51.6"N and 9°22′28.05"N and longitudes 33°0′24.4"E and 38°24′42.24"E. The total area of the catchment is about 79,000 km^2^ with a length of 550 km and an average width of 143.64 km [48]. The area is drained by some of the major rivers of the country, such as the Omo, Gilgel Gibe, Gojeb, and their numerous tributaries. These have created the dissected terrain. Nearly half of the country’s remaining natural forests are found in this region [49]. It is an enclosed river basin that flows into Lake Turkana in Kenya, forming its southern boundary. The western watershed ranges from hills and mountains that separate the Omo Gibe basin from the Baro-Akobo basin. To the north and northwest, the basin is bounded by the Blue Nile basin with a small area in the northeast bordering the Awash basin [48].The mean annual rainfall and temperature of the basin are 1425 mm and 19.2°C, respectively [50].

### Spatial mapping of sampling sites

A Digital Elevation Model (DEM) data and Sentinel-2 images of the study area were downloaded from the United States Geological Survey website (https://earthexplorer.usgs.gov) from which altitudinal difference and Land Use/Land Cover (LULC) were computed. Sentinel-2 images with a spatial resolution of 10 m were used to assess LULC of the study area through Earth Resource Data Analysis System (ERDAS) 2015 image processing software. Images used dated from the dry season of 2019. Prior to image analysis, initial processing on the raw data was carried out to correct for any distortion due to the characteristics of the imaging system and conditions. In pre-processing of images; layer stacking (band composite), mosaic of different swath images and sub-setting of an image to the study area were done using ERDAS image 2015 to form a different combination of red, green, blue color composition. Supervised classification was carried out for identifying LULC types in the study area. Among different algorithms in the supervised classification, maximum likelihood, which assumes that each spectral class can be described by a multivariate normal distribution, was utilized. For this supervised image classification, training areas were established based on the ground control point taken during fieldwork. The map templates of different LULC types were computed from these satellite images. The catchment landscape (LULC around sampling sites) was classified into five categories, including forest, shrub, wetland, farmland and settlement area based on the guideline of the Food and Agriculture Organization (FAO) [51]. Forest are areas with trees reaching 5 m in height, greater or equal to 0.5 ha in area and a canopy cover of >10% without other land use. Shrub are areas covered with dense or scattered grasses and wood, shrubs and trees. Wetland is plain land along rivers characterized by the presence of emergent aquatic plants. Farmland are areas characterized by the presence of any agricultural crops and bare ground that had been prepared for planting crops. Dispersed rural settlements and homesteads were classified as settlement areas [51].

To assess the classification accuracy, a confusion matrix was employed. Accuracy of the classified LULC maps was assessed using a combination of overall accuracy, producer’s accuracy, user’s accuracy, errors of commission and omission [52] and kappa coefficient [53]. Hence, the overall accuracy of satellite image classification in the study area is 87.33% with a Kappa index agreement of 0.8492.

**Fig 2:**
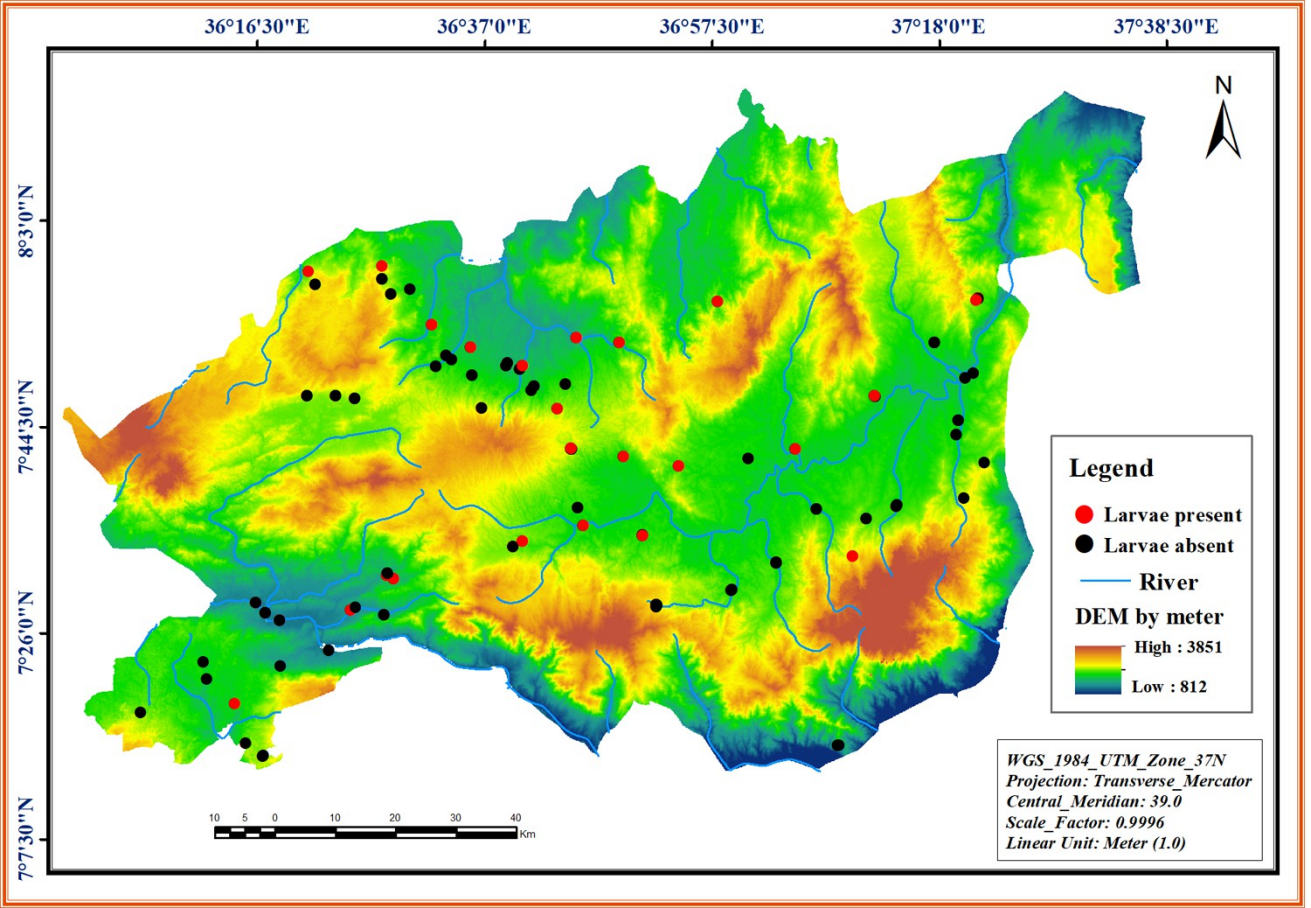
Digital Elevation Model (DEM) image of Omo Gibe river basin (southwest Ethiopia) indicating locations of the sampling sites.

The study area and LULC were mapped and visually digitized using the satellite image in the Geographic Information System (GIS) software packages ERDAS 2015, ArcGIS 10.7 and validated by ground truth points. The DEM image of the study area derived from Advanced Spaceborne Thermal Emission and Reflection Radiometer of 30 m re-sampled to 10 m spatial resolution is given in **Fig 2**.

Both environmental (physico-chemical water quality parameters and ecological habitat characteristics) and biological (blackfly larvae and aquatic macroinvertebrate predators) data were collected during both dry (April) and wet (November) seasons in 2019. Data were collected from 150 sampling sites in the Omo Gibe river basin of southwest Ethiopia. Sampling sites were selected according to their accessibility and the presence of water flow.

### Environmental variables

Measurements of environmental variables were carried out both in the field and in the laboratory. Physico-chemical water quality parameters, such as water temperature, dissolved oxygen, pH and electrical conductivity, were measured onsite using a portable Multi-Probe Meter (HQ40d Single-Input Multi-parameter Digital Meter; Hach Company, Loveland, USA). Water turbidity was measured using a Turbidity Meter (Wag-WT3020; Halma PLC Company, Amersham, UK). Total dissolved solids were measured using a Palintest (Palintest Photometer 800 Field Kit; Camlab Limited, Wigan, UK). A water sample (2000 ml) was collected from each sampling site in polyethylene bottles and transported to the laboratory using an ice cooler box for analysis of other physico-chemical parameters. A water sample (250 ml) was filtered through a 0.45 μm filter paper and then analyzed for concentrations of alkalinity, total hardness, nitrate, ammonia, orthophosphate and ions such as chloride, calcium and magnesium. An unfiltered water sample was used to determine the concentrations of total suspended solids, five-day Biological Oxygen Demand (BOD_5_) and total phosphorus. These analyses were carried out according to the standard method [54] at the laboratory of Environmental Health Sciences and Technology of Jimma University.

Ecological habitat characteristics such as water depth, water body width, flow velocity and flow rate were determined at each sampling site using a standard protocol [55]. Water depth was determined with the aid of a labeled rod dipped vertically into the stream until the streambed was reached, thereafter withdrawn and then the reading was recorded. Meter tape was used to measure the width of the water body. A cork and a timer watch were used to measure surface water flow velocity; the time taken for a cork to move one meter in the distance was used to determine the velocity [56]. Water flow velocity, water depth and water width measurements were used to estimate the flow rate. Classification of riparian vegetation cover (open, brush, and forest), canopy cover (none, partial and complete) and dominant streambed particle size (silt, sand, gravel, cobble, boulder and bedrock) were made following the protocol by McCreadie et al. [56].

At each sampling site, altitude and point coordinates (latitude and longitude) were taken using a hand-held global positioning system instrument (GPS 72H; Garmin Ltd., Olathe, Kansas, USA).

### Sampling and identification of *S*. *damnosum* larvae

Blackfly larvae were sampled using a standard hand-held rectangular drag (20×30 cm) with a mesh net of 300 μm [57]. A stretch of 10 m distance of the stream flow was sampled for approximately 10 min. Blackfly larvae were collected from all available substrates by hand using fine forceps. Specimens were preserved in Carnoy’s solution (acetic acid: ethanol, 1:3) and transported to laboratory for taxonomic identification. Only the matured larvae (showing dark gill spots) were morphologically identified at species level using a stereomicroscope (10x) and taxonomic identification keys for larvae of the African *Simulium* [58].

### Sampling and identification of blackfly predators

Aquatic macroinvertebrates (other than blackfly larvae) were sampled from the same habitat where blackfly larvae were sampled. The macroinvertebrate sampling was carried out using a standard hand-held rectangular drag (20×30 cm) with a cutting metal frame covered with a mesh net of 300 μm according to the method described by Gabriels et al. [57]. Collected specimens were sorted in the field, preserved in 80% ethanol and transported to laboratory for taxonomic identification. In the laboratory, the macroinvertebrates were identified at a family level using a stereomicroscope (10×) and identification keys [55]. Macroinvertebrate families including Chironomidae [43, 46, 59], Hydrometridae [39], Hydropsychidae [44, 45], Perlidae [42], Gomphidae, Aeshnidae, Libellulidae, Coenagrionidae [41, 45, 46], Baetidae [46] and Dytiscidae [46] have been reported as potential predators of immature blackflies. Therefore, in this study, the presence or absence (1/0) and abundance of these macroinvertebrate predators were considered as biotic factors to assess the occurrence and abundance of blackfly larvae.

### Data analysis

The abundance of *S*. *damnosum* s.l. (hereafter *S*. *damnosum*) larvae did not follow a normal distribution (tested with Shapiro-Wilk Normality test). Therefore, a non-parametric Kruskal*-*Wallis Analysis of Variance (ANOVA) was used to analyze the variation in the larval abundance among ecological habitat characteristics (i.e., type of canopy cover, type of riparian vegetation cover and streambed particle size). If significant differences were observed in Kruskal*-*Wallis ANOVA, a Wilcoxon *post-hoc* multiple comparison test was used to identify significantly different pairs. The *post*-*hoc* test was Bonferroni corrected. A Wilcoxon Rank-Sum test was also used to compare the abundance of blackfly larvae between seasons. In addition, the measurements of environmental variables of habitats with blackfly larvae were compared with those of habitats without blackfly larvae using a Wilcoxon Rank-Sum test.

#### Generalized linear model

Generalized linear models (GLMs) were applied to determine the habitat preference of *S*.*damnosum* larvae based on environmental variables. In addition, separate GLMs were applied to determine the influence of macroinvertebrate predators on the occurrence and abundance of *S*.*damnosum* larvae. A GLM with logistic regression was applied to predict the occurrence of *S*. *damnosum* larvae, while a GLM with negative binomial regression was employed to predict the abundance of *S*. *damnosum* larvae. In the initial step of developing the models, the dataset was explored to detect outliers and collinearity among all predictor variables to decrease the uncertainty of the model [60]. To find a set of predictors that do not contain collinearity, Spearman’s rank-order correlation coefficient was determined to generate a matrix of pairwise correlations between all the predictor variables. As correlation coefficients only show pairwise correlations, we calculated Variance Inflation Factors (VIFs) to assess which predictor variables are collinear and should be dropped before starting the analyses. This procedure continued until no further collinearity existed [60].

A stepwise backward selection procedure was followed to build the model starting from the full model. The model with the lowest Akaike Information Criterion (AIC) value was retained as the optimal model [60]. The goodness-of-fit of the models was assessed using the relations between the residuals (the differences between observations and predictions by the retained model) and predictor variables. The normality of the residuals was tested using a QQ-plot (probability plot). Retained models were only considered reliable if no relations between the residuals and the predictor variables were visually observed and residuals were normally distributed [60]; the retained models were rejected otherwise. An alpha value of *p* < 0.05 was considered statistically significant. Data exploration and regression modeling were performed using R software (Version 3.5.2) [61].

#### Multivariate analysis

Relationships between the environmental and biological (*S*. *damnosum* larvae and blackfly predators) data were examined using multivariate analysis with the software program CANOCO for Windows version 4.5 [62]. Detrended Correspondence Analysis (DCA) was used to determine the appropriate response (linear or unimodal) for biological data. The performed DCA yielded a length of gradient greater than 2 standard deviations, implying that the biological data exhibit a unimodal type of response along environmental gradients. Therefore, we used Canonical Correspondence Analysis (CCA) for data analysis. Prior to data analysis, when two or more variables had a VIF greater than 5, one of these variables was removed from the analysis. AVIF of 5 and greater has been identified as an indicator of collinearity in multivariate analysis [63]. Biological and environmental data, except pH, were log-transformed [(log (x + 1)] to improve normality and homoscedasticity.

A stepwise forward selection was employed to identify the smallest set of statistically significant variables that contribute most to the explained variance in the response variables. The statistical significance of eigenvalues and taxa-environment correlations generated by the CCA were tested using a Monte Carlo test with 999 permutations [62].

## Results

### Occurrence and abundance of blackfly larvae

The only blackfly species that was collected during the study period was *S*. *damnosum*. A total of 5927 *S*. *damnosum* larvae were collected, of which 3345 (56%) individuals were collected in the wet season and 2582 (44%) in the dry season. The number of *S*. *damnosum* larvae per sampling site for all collections varied from 0 to 283 individuals.

### Spatial distribution of blackfly larvae

**Fig 3:**
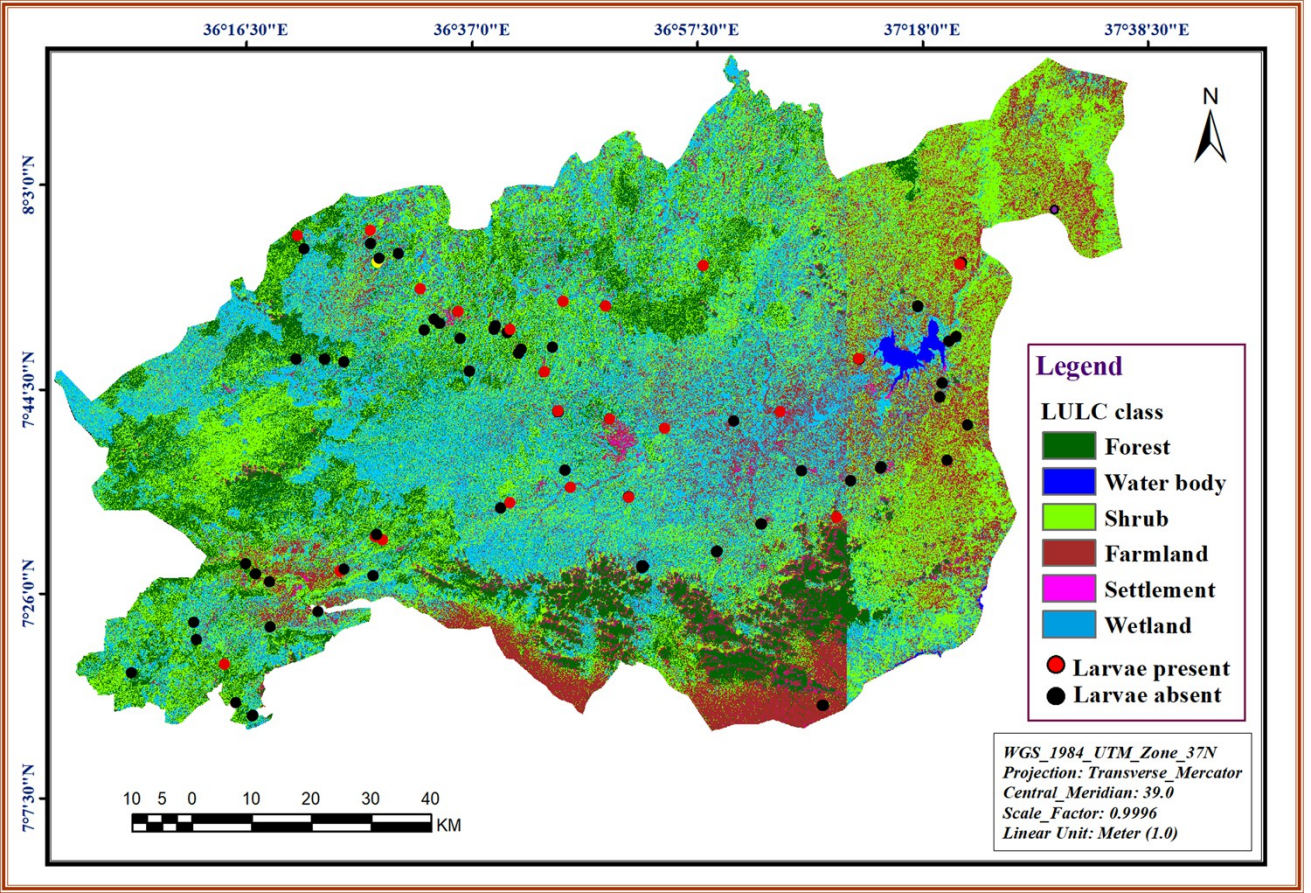
Spatial distribution of habitats of *S*. *damnosum* larvae in the Omo Gibe river basin, southwest Ethiopia.

The spatial mapping for sampling sites indicated that habitats of *S*. *damnosum* larvae were distributed across all LULC types in the study area. *Simulium damnosum* larvae more likely present in habitats near shrub, whereas less likely present in habitats near human settlement (**Fig 3**). Highest abundance of *S*. *damnosum* larvae was collected from habitats with riparian forest cover (*p* < 0.05). There was no statistically significant difference in the abundance of *S*. *damnosum* larvae between seasons as well as among types of canopy cover and types of streambed particle size (all *p* > 0.05).

### Importance of environmental factors

A summary of environmental variables used to determine the habitat preference of *S*. *damnosum* larvae is given in **Table 1**. The concentrations of BOD_5_, total phosphorus and orthophosphate were higher at habitat without *S*. *damnosum* larvae compared to those with *S*. *damnosum* larvae (*p <* 0.05). Statistically significant associations were observed between nitrate and ammonia, between calcium ion and total hardness, between magnesium ion and total hardness and between water width and flow rate all *p* < 0.05). Total hardness, nitrate and flow rate were included in the model development as they have been reported as important variables affecting the population of *S*. *damnosum* larvae [35, 64].

**Table 1:** Descriptive statistics of environmental variables used to determine the habitat preference of *S*. *damnosum* larvae in the Omo Giber river basin, southwest Ethiopia.

Environmental variable	Unit	Minimum	Maximum	Median	Mean	SD
Water temperature	°C	12	28	23	22.6	3
Dissolved oxygen	mg/l	5	8	7	6.9	0.6
Oxygen saturation	%	63	110	97	94.4	9
BOD_5_	mg/l	0.1	7	2	2.2	2
pH	-	6	10	7	7.2	0.5
Turbidity	NTU	6	516	34	62.1	88
Electrical conductivity	μS/cm	9	176	72	73	33
Total dissolved solids	mg/l	6	120	50	53.8	22
Total suspended solids	mg/l	0.8	723	44	67.6	91
Alkalinity	mg/l	0	124	27	25.4	20
Nitrate	mg/l	0	8	0.1	0.5	2
Ammonia	mg/l	0	22	0.1	1.6	5
Orthophosphate	mg/l	0.1	15	0.4	0.6	1
Total phosphorus	mg/l	0.1	64	0.9	2.8	7
Chloride	mg/l	2	40	11	11	7
Calcium	mg/l	0	68	20	20	11
Magnesium	mg/l	0	59	8	11.8	13
Total hardness	mg/l	0	90	28	31.8	20
Water depth	M	0.1	2.4	0.4	0.5	0.4
Water width	M	0.1	50	2	5	8
Flow velocity	m/s	0.1	2	0.6	0.7	0.4
Flow rate	m^3^/s	0.1	24	0.4	2.3	5
Altitude	M	1306	2198	1749	1752	193
Canopy cover	Category	N/A	N/A	N/A	N/A	N/A
Riparian vegetation cover	Category	N/A	N/A	N/A	N/A	N/A
Streambed particle size	Category	N/A	N/A	N/A	N/A	N/A
LULC type	Category	N/A	N/A	N/A	N/A	N/A

*Abbreviation*: NTU, nephelometric turbidity unit; SD, standard deviation; N/A, not applicable.

The results of the logistic regression analysis indicated the presence of *S*. *damnosum* larvae was positively associated with electrical conductivity and altitude, whereas negatively associated with total hardness and water depth. According to the negative binomial regression analysis, the abundance of *S*. *damnosum* larvae increased with increasing turbidity, alkalinity and altitude, but decreased with increasing concentrations of BOD_5_ and orthophosphate. Habitats characterized by boulders, cobbles and silty streambed particles supported abundant *S*. *damnosum* larvae (**S1 Table**).

### Relationship between blackfly larvae and macroinvertebrate predators

A total of 10 families of potential macroinvertebrate predators of the blackfly larvae belonging to 7 orders were collected (**Table 2**).

**Table 2:** Potential macroinvertebrate predators of blackfly larvae collected from streams in the Omo Gibe river basin, southwest Ethiopia.

Order	Family	Frequency of occurrence (%)	Relative abundance (%)
Odonata	Aeshnidae	10	1
	Gomphidae	30	4
	Libellulidae	43	4
	Coenagrionidae	34	4
Trichoptera	Hydropsychidae	89	40
Ephemeroptera	Baetidae	83	36
Plecoptera	Perlidae	20	2
Coleoptera	Dytiscidae	5	0
Hemiptera	Hydrometridae	2	0
Diptera	Chironomidae	46	9

According to the output of the regression models, the presence and abundance of S. *damnosum* larvae were negatively affected by the abundance of stonefly larvae (Perlidae). *Simulium damnosum* larvae are less likely present in the presence of mayfly larvae (Baetidae) and were less abundant where Chironomidae is abundant. In contrast, both the presence and abundance of *S*. *damnosum* larvae were positively affected by the presence of caddisfly larvae (Hydropsychidae) (**S2 Table**).

### Ordination analysis

**Fig 4:**
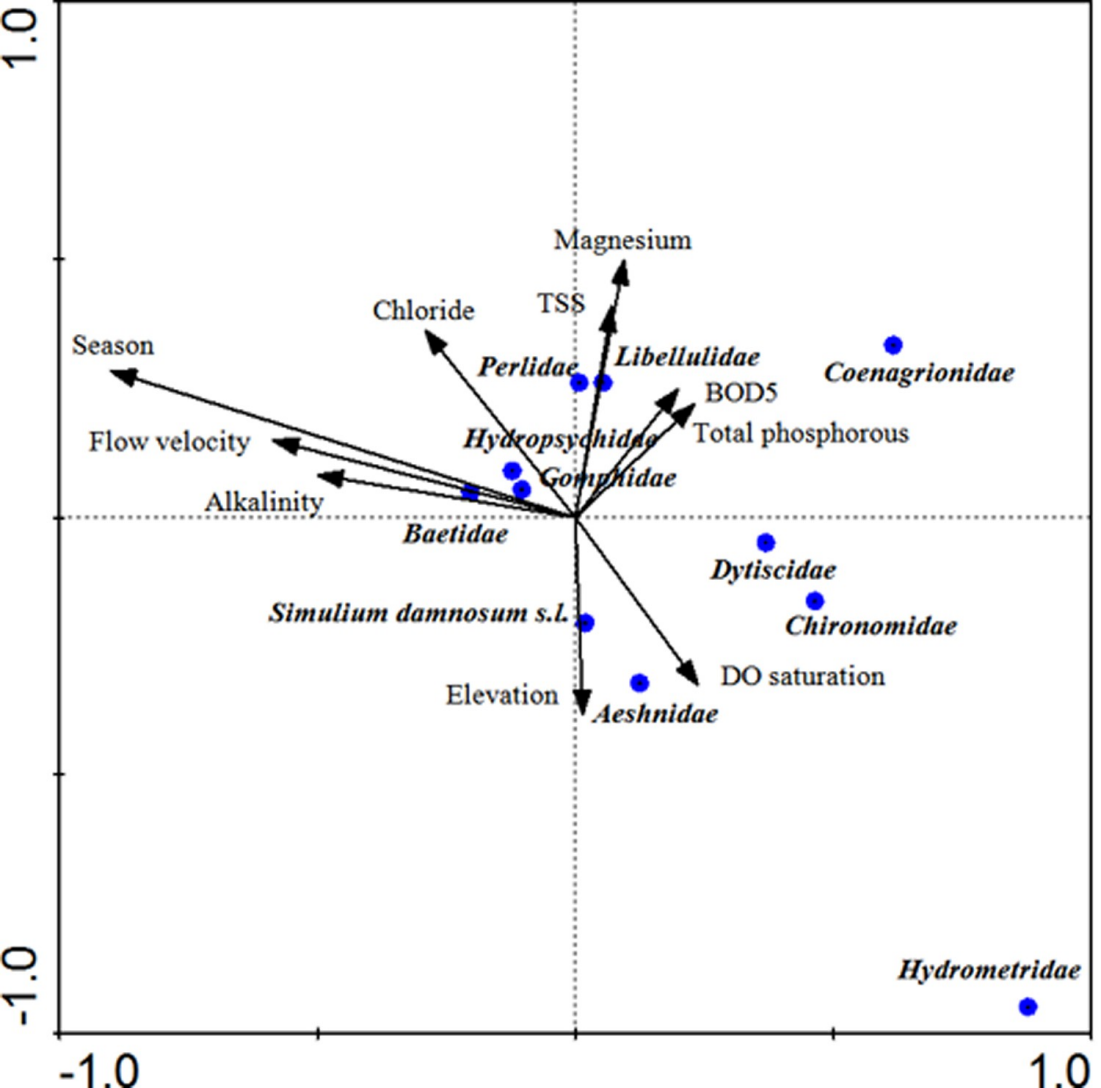
Canonical Correspondence Analysis (CCA) of *S*. *damnosum* larvae and biological and environmental variables in Omo Gibe river basin. pH: Logarithmic measure of hydrogen ion concentration; DO: Dissolved Oxygen; TSS: Total Suspended Solids; TDS: Total Dissolved Solids; BOD5: Five-day Biological Oxygen Demand.

The first and the second canonical axes explained 16.1% and 8% of the variances in the biological data, respectively. The taxa-environment correlations of the first two axes were statistically significant in a Monte Carlo permutation test (*p* < 0.05). In this ordination, the taxa-environment correlation for the first two axes was 0.72 and 0.59, respectively. The first axis of the CCA ordination revealed a gradient primarily associated with the season. The first axis of the CCA was positively correlated with flow velocity but negatively correlated with electrical conductivity. The second axis of the CCA was positively correlated with BOD_5_ and it was negatively correlated with alkalinity (**Fig 4**).

## Discussion

The findings of this study revealed that the habitat preference of *S*. *damnosum* larvae is determined by a wide range of environmental factors. For instance, the abundance of *S*. *damnosum* larvae increased with increasing alkalinity, turbidity and electrical conductivity of water. The high levels of turbidity, alkalinity and electrical conductivity of water recorded in habitats of blackfly larvae could be attributed to runoff from nearby land use in the catchment area [37, 65, 66]. On the other hand, *S*. *damnosum* larvae were less abundant in habitats with high BOD_5_ and orthophosphate contents which matches with earlier finding by Bernotiene [64]. Besides the variables reported in this study other water quality variables (water temperature, flow velocity, pH, total suspended solids, total dissolved solids and orthophosphate) have shown to affect the presence and abundance of blackfly larvae in previous studies [5, 29, 33, 37].

Previous studies have also reported that altitude is an important factor determining the presence and abundance of blackfly larvae [5, 67, 68]. Our finding also indicated that the presence and abundance of *S*. *damnosum* larvae increased with increasing altitude. This could be related to an increase in flow velocity of water with an increasing slope of the land surface along altitudinal gradients, which in turn increases dissolved oxygen concentration in water. Moreover, the possible explanation for this finding could be due to the reduction in diversity and density of blackfly predators with increasing altitude [69–71]. We also noted a decrease in densities of blackfly predators (i.e., Baetidae, Hydropsychidae) with increasing altitude in the Omo Gibe river basin. The abundance of *S*. *damnosum* larvae in habitats characterized by boulders, cobbles and silty streambed particles observed in our study indicates the availability of food sources and the accessibility of suitable surfaces for the attachment of blackfly larvae. Our observation corroborates with earlier observations [72–74]. The results of our study also indicated that *S*.*damnosum*. larvae were abundant in habitats with riparian forest cover which might be due to the availability of plant leaves as a source of food and shelter [75–78].

Freshwater ecologists have reported strong links between the occurrence of blackfly larvae and macroinvertebrate communities; which could be an indication for their predominant role in biocontrol of blackflies via predation and/or competition [29, 38, 39, 41]. Similar observations have been made in our study showing both the presence and abundance of *S*. *damnosum* larvae were negatively affected by the abundance of stonefly larvae (Perlidae). Moreover, *S*. *damnosum* larvae less likely present in the presence of mayfly larvae (Baetidae).Several studies have confirmed predation of blackfly larvae by stonefly (Perlidae) and mayfly (Baetidae) which were detected through stomach dissection and serological tests [42, 46, 79]. The occurrence and abundance of blackfly larvae are affected not only by predation but also by competition for food sources [80].

Dipterans are usually regarded as predators of blackfly larvae in previous studies [46, 59]. The decrease in presence and abundance of *S*. *damnosum* larvae with increasing abundance of Chironomidae observed in our study might not be only prey-predator interaction, which could be due to the difference in habitat preferences of the two taxa. Blackfly larvae are associated with well-oxygenated running water [5], whereas Chironomidae is known as pollution tolerant taxa commonly found in a degraded watershed [81, 82].

Trichopterans have been confirmed to be a blackfly larvae predator in literature [44, 45]. On the contrary, our study showed that the presence and abundance of *S*. *damnosum* larvae increased in the presence of caddisfly larvae (Hydropsychidae), which might be due to different drivers other than predation. For instance, caddisfly larvae (Hydropsychidae) are opportunistic generalist predators [44, 45, 83] and thus they can change their feeding behavior and choose between hunting, grazing, and catching drifting food with their nets [84].The abundance of organisms in aquatic ecosystems is not only related to the presence of predators but also to the traits that aid in predator defense [85]. Many aquatic organisms are capable of altering their behavior and morphology to reduce predation rates in the presence of predators [86]. Moreover, breeding habitat characteristics such as streambed composition can provide refuges that alleviate the negative impacts of the predators [87].

Potential macroinvertebrate predators and competitors of blackfly larvae, including the blackfly itself, are important pollution indicators of running water and they are known for their low pollution tolerance [88]. This indicates that the blackfly larvae and their potential biocontrol agents respond to the deterioration of aquatic environments in a similar way [88, 89]. Aquatic environments in the Omo Giber river basin are at risk of pollution due to anthropogenic activities in the catchment areas [90–92]. Fertilizer residues can runoff into adjacent rivers and cause eutrophication of the rivers, which can lead to a decrease in water quality and loss of biodiversity including biocontrol agents of blackflies [89, 93]. Insecticides and herbicides applied to agricultural land can also enter rivers, thereby adversely impact aquatic organisms [94, 95]. In general, deterioration of aquatic ecosystems due to anthropogenic activities can adversely affect the macroinvertebrate assemblages including the potential biocontrol agents of immature blackflies. Therefore, it is essential to protect aquatic environments from pollution to boost biodiversity which is intended to conserve the assemblages of macroinvertebrate predators (density and species) that are already present within the system and enhance the opportunity of prey-predator interactions.

In conclusion, the habitat preference of *S*. *damnosum* larvae is determined by environmental factors. The information obtained from this study also offers important insights that the presence and abundance of *S*. *damnosum* larvae are affected by macroinvertebrate predators, implying that they have the potential to be used as biocontrol agents of immature blackflies to enhance onchocerciasis elimination efforts. Hence, it is essential to establish buffer zones as a part of watershed management to retain pollutants and prevent them from entering directly into watercourses to improve water quality and the assemblages of macroinvertebrate predators and hence, to enhance biocontrol of blackflies.

As a limitation of the study, the macroinvertebrate predators identified at the family level may not entirely reflect the influence of specific predator species on the population of blackfly larvae. Therefore, further study is required to understand the influence of the predator species on the occurrence and abundance of blackfly larvae.

## Supporting information

S1 Table Output of GLM to determine the habitat preference of *S*. *damnosum* larvae based on environmental variables. (DOCX)

S2 Table Output of GLM to investigate the relationship between *S*. *damnosum* larvae and macroinvertebrate predators. (DOCX)

S1 Data (XLSX)
