# Supplementary material for: Habitat preference of blackflies in Omo Gibe river basin (southwest Ethiopia): Implications for onchocerciasis elimination and control
Source: PLoS One. 2022 Mar 4;17(3):e0264750. doi: 10.1371/journal.pone.0264750 (PMC8896702; doi:10.1371/journal.pone.0264750)
Supplement: S1 Table — (DOCX) [file pone.0264750.s001.docx]

| ***S. damnosum* larvae** | **Environmental variables** |  | **Estimate** | **Std. error** | **Z value** | **Pr(>\|z\|)** |
| --- | --- | --- | --- | --- | --- | --- |
| Occurrence | Total hardness |  | -2.749e-02 | 1.323e-02 | -2.077 | 0.037764 * |
|  | Electrical conductivity |  | 2.067e-02 | 1.004e-02 | 2.059 | 0.039466 * |
|  | Water depth |  | -1.455e+00 | 7.300e-01 | -1.993 | 0.046285 * |
|  | Elevation |  | 4.793e-03 | 1.449e-03 | 3.308 | 0.000941 *** |
|  | Riparian vegetation cover | Forest | 1.600e+00 | 6.394e-01 | 2.502 | 0.012360 * |
|  |  | Bush |  |  |  |  |
|  |  | Open | 1.729e+00 | 8.450e-01 | 2.046 | 0.040786 * |
| Abundance | BOD_5_ |  | -1.681e-01 | 8.356e-02 | -2.012 | 0.0442 * |
|  | Turbidity |  | 4.022e-03 | 2.012e-03 | 1.999 | 0.0456 * |
|  | Alkalinity |  | 1.805e-02 | 7.810e-03 | 2.311 | 0.0208 * |
|  | Orthophospahte |  | -1.318e+00 | 6.116e-01 | -2.155 | 0.0311 * |
|  | Elevation |  | 1.528e-03 | 7.527e-04 | 2.030 | 0.0424 * |
|  | Streambed particle size | Silt | 2.800e+00 | 1.151e+00 | 2.432 | 0.0150 * |
|  |  | Sand | 9.894e-01 | 1.290e+00 | 0.767 | 0.4430 |
|  |  | Gravel |  |  |  |  |
|  |  | Cobble | 2.089e+00 | 1.005e+00 | 2.079 | 0.0376 * |
|  |  | Boulder | 2.291e+00 | 9.910e-01 | 2.312 | 0.0208 * |
|  |  | Bedrock |  |  |  |  |

Signif.codes: 0 ‘***’ 0.001 ‘**’ 0.01 ‘*’ 0.05 ‘.’ 0.1 ‘ ’ 1
