# Supplementary material for: Habitat preference of blackflies in Omo Gibe river basin (southwest Ethiopia): Implications for onchocerciasis elimination and control
Source: PLoS One. 2022 Mar 4;17(3):e0264750. doi: 10.1371/journal.pone.0264750 (PMC8896702; doi:10.1371/journal.pone.0264750)
Supplement: S2 Table — (DOCX) [file pone.0264750.s002.docx]

| ***S. damnosum* larvae** | **Biological variables** | **Estimate** | **Std. error** | **Z value** | **Pr(>\|z\|)** |
| --- | --- | --- | --- | --- | --- |
| Occurrence | Perlidae abundance | -0.16137 | 0.06319 | -2.554 | 0.011 * |
|  | Chironomidae abundance | -0.03518 | 0.01719 | -2.047 | 0.041 * |
|  | Baetidae presence | -1.63219 | 0.77873 | -2.096 | 0.036 * |
|  | Hydropsychidae presence | 1.91573 | 0.76869 | 2.492 | 0.013 * |
| Abundance | Perlidae abundance | -0.16235 | 0.04604 | -3.526 | 0.000 *** |
|  | Perlidae presence | 1.06760 | 0.44138 | 2.419 | 0.0156 * |
|  | Hydropsychidae presence | 1.72237 | 0.52819 | 3.261 | 0.001 ** |

Signif.codes: 0 ‘***’ 0.001 ‘**’ 0.01 ‘*’ 0.05 ‘.’ 0.1 ‘ ’ 1
